# Relationship Bonds and Service Provider’s Emotional Labor: Moderating Effects of Collectivism

**DOI:** 10.3389/fpsyg.2019.00370

**Published:** 2019-02-26

**Authors:** Myoung-Soung Lee, Sang-Lin Han, Suji Hong, Hyowon Hyun

**Affiliations:** School of Business, Hanyang University, Seoul, South Korea

**Keywords:** relationship bonds, emotional labor, person-organization fit, collectivism, service provider

## Abstract

Since service providers directly conduct emotional labor to customers, it is important to identify the factors influencing emotional labor of service providers. Even though the studies identifying the predisposing factors influencing emotional labor are taking place, there is no empirical evidence confirming how relationship bonds, which have been established between corporations and service providers, are related to emotional labor. This study examined the influences of relationship bonds on emotional labor through person-organization fit (P-O fit) and the moderating effects of collectivism between P-O fit and emotional labor. Analysis was conducted by performing questionnaire surveys targeting 350 employees in the financial industry. As a result of the analysis, it has been found that financial bonds, social bonds, and structural bonds enhanced P-O fit and P-O fit improved deep acting. In addition, this study identified that collectivism of service providers strengthened the influence of P-O fit toward deep acting. This study not only suggested the empirical evidence identifying the process of relationship bonds influencing emotional labor but also expanded the scope of study by examining moderating roles of collectivism in cultural psychology aspect.

## Introduction

Emotional labor is a service provider’s effort to present emotions in a way that is desired by the organization (Meier et al., 2006) and a form of emotion control which creates a publicly visible facial and bodily display in the workplace (Hochschild, 1983). Therefore, service providers engaged in emotional labor try to induce customers’ positive responses with the display of appropriate emotions such as smile by controlling personal emotion (Wang, 2014). Such emotional labor is closely related with service providers as it happens mainly during the interaction between customers and service providers on behalf of an organization (Ashforth and Humphrey, 1993).

Many of the preceding studies on emotional labor focused on customer response to the emotional labor (Hennig-Thurau et al., 2006; Groth et al., 2009), or the influence of emotional labor on the employee’s job outcomes (Ashforth and Humphrey, 1993; Van Dijk and Brown, 2006). Not a few researchers confirmed that service provider’s display of positive emotion enhances customer evaluation about service quality (Pugh, 2001), increases reuse intentions (Wang, 2009), and leads to positive word-of-mouth (Wang, 2014). From the perspective of corporations, emotional labor of the service provider leads to positive results. From the service provider’s viewpoint, however, it can cause negative outcomes such as an increase of job stress and sick leave and high turn-over rate (Hatzinikolakis and Crossman, 2010).

Affective delivery means the delivery of emotion to the customer by service provider (Christoforou and Ashforth, 2015). Affective delivery is associated with emotional exhaustion (Trougakos et al., 2008). Emotional labor requires more emotional resources than affective delivery and causes a higher level of emotional exhaustion (Smith et al., 2009). If corporations neglect emotional labor and job stress of service providers, they are exposed to a bigger risk of emotional exhaustion which can lead to higher turnover and lower commitment to the organization (Huang and Dai, 2010). In that sense, it is important for firms to understand the factors influencing emotional labor of service providers but there have been only few studies regarding the antecedent factors of emotional labor. Furthermore, previous studies have been conducted with regard to the factors influencing emotional labor based on the factors related to job and individual characteristics (Hur et al., 2014). Job autonomy (Diefendorff and Gosserand, 2003), emotional requirement (Grandey, 2003), time pressure (Ashforth and Humphrey, 1993) have been identified as the leading factors related to job influencing emotional labor. Some important issues with regard to individual characteristics, the effects of emotional intelligence (Delpechitre and Beeler, 2018), chronological age and work experience (Hur et al., 2014), demographic factor (Schaubroeck and Jones, 2000) of service providers have been identified. Even though scope of study on emotional labor is being expanded, researches on predisposing factors of emotional labor are very limited.

One of the factors influencing service provider’s emotional labor is related to the organization which they belong to. As their perception about the organization influences the emotional labor (Yoo and Arnold, 2016), businesses need to make an effort to have a positive relationship with the service providers working for them. Studies on relationship marketing suggest relationship bonds as strategy for a corporation to build a relationship with customers which proved crucial for better relationship performance (Dash et al., 2009). Most of the existing research on relationship bond focused on strategies to build ties between corporations and customers, or between corporations, and studies from the perspective of employees has not been adequate. Accordingly, this study aims to identify the influences of the relationship bond on the emotional labor of service providers.

Relationship between a corporation and service providers positively influences the service provider’s affective commitment and normative commitment to the corporations (Wang, 2014), which in turn enhances person-organization fit. Conversely, this person-organization fit can influence the emotional labor of the service providers. When individuals perceive organizational characteristics or values consistent with their own, they tend to show positive emotions and attitude toward the organization but when they perceive the opposite, they are more likely to be negative in their emotions and attitude due to conflicting values (Cable and Judge, 1996; Lauver and Kristof-Brown, 2001). As value conflicts between individuals and organization tend to require more emotional resources, poor person-organization fit affects negatively emotional labor. On the other hand, cultural propensity of an individual is another factor influencing emotional labor. Employees with collectivistic tendencies are more likely to be cooperative as they put more emphasis on the relationship with the group than on personal needs (Ozdemir and Hewett, 2010). This indicates that service providers with higher collectivist tendencies are more actively engaged in emotional labor to achieve corporate goals. Therefore, collectivism can moderate the influence of person-organization fit (P-O fit) on emotional labor.

This study aims to examine the influences of relationship bonds on emotional labor through person-organization fit: The influence of the three elements of relationship bond (financial bonds, social bonds, and structural bonds) on P-O fit of service providers, the influence of P-O fit of service providers on their emotional labor, and moderating effects of collectivism between P-O fit and emotional labor.

## Theoretical Background and Research Hypotheses

### Emotional Labor

Emotional labor, using employees’ emotions to enhance goals of firms (Grandey and Melloy, 2017), is the effort to expose the emotions actually felt by employees as the emotions wanted by customers after regulating and managing them (Grandey, 2000; Molino et al., 2016). Corporations set employees’ emotions and the expression modes toward customers and employees moderate their own emotions by conforming to emotional display rules (Ashforth and Humphrey, 1993). Such an emotional labor has a lot to do with service providers who interact with customers instead of corporations (Ashforth and Humphrey, 1993). Since service providers not only perform work while interacting with customers but also become a part of outcomes of expressing emotions, moderating one’s emotions is direct and essential. Thus, emotional labor of service providers is higher than that of other office workers or physical laborers (Hochschild, 1983; Grandey and Melloy, 2017).

Previous studies divided emotional labor strategies into surface acting and deep acting (Grandey, 2000; Yoo and Arnold, 2016). Surface acting is expressing emotions after matching them with emotional display rules required by corporations (van Gelderen et al., 2017) and deep acting is matching individual’s emotions with emotional display rules required by corporations (Hennig-Thurau et al., 2006). In other words, with regard to surface acting, emotions are suppressed and expressed deceivingly in order to conduct emotional display rules (Ashforth and Humphrey, 1993) and with regard to deep acting, emotions to be expressed to customers are internalized and efforts are made to actually feel them (Yoo and Arnold, 2016). Since service providers must express emotions according to emotional display rules, they have to follow the rules after choosing either surface acting or deep acting (Hochschild, 1983). Therefore, corporations should induce service providers to do deep acting. If service providers do express the emotions required by corporations but feel different emotions, they are stressed from emotional discord. On the other hand, if service providers feel the emotions which must be expressed to customers, emotional discord is removed and positive psychological result is brought about (Brotheridge and Grandey, 2002). In that sense, the factors influencing emotional labor of service providers must be identified.

### Relationship Bonds

Relationship bonds refers to corporations more effectively reaching stakeholders and forming long-term and continuous relationships (Copulsky and Wolf, 1990; Wu and Lin, 2014). Through such relationship bonds, stakeholders make a commitment to the relationships with corporations since they can get continuous outcomes (Wang, 2014).

Previous studies divided the relationship bonds into financial bonds, social bonds, and structural bonds based on characteristics of relationship bonds, customization, and degree of relationship sustainability (Berry, 1995; Kim and Kim, 2018). Financial bonds refer to providing financial benefits through the most fundamental relationship formation strategy (Berry, 1995) and some examples are corporations providing incentives to customers and striving to form relationships through discounts (Hsieh et al., 2005). Similarly, relationships can be formed with stakeholders other than customers through financial dependence. Financial incentives such as salary become financial bonds to service providers (Wang, 2014) and degree of financial bonds is decided based on level of salary.

Social bond is based on establishing close ties among stakeholders through social interaction or friendship (Liang and Wang, 2005) and they can acquire psychological gains through it (Gwinner et al., 1998). Moreover, establishing relationship through interaction creates emotional engagement among stakeholders and makes no replacing relationship by another target (Lee et al., 2015). Service providers establish and progress relationships through the interaction with other colleagues (Gremler and Gwinner, 2000). Also, interaction among service providers provides psychological benefits by forming intimacy and trust (Rodríguez and Wilson, 2002). Service providers who have established positive social relations become emotionally immersed in that corporation and have the will to belong to it continuously (Wang, 2014). Therefore, from the standpoint of service providers, social bond is the corporate effort to establish social relations.

Structural bond refers to create the value stakeholders need (Wang, 2014). This means providing unique and customized values which cannot be obtained from others and these customized values are generated when the right to make decisions is given to stakeholders (Bolton et al., 2003). Giving the right to stakeholders to make decisions by themselves – the arrangement of the opportunities to receive the services tailored to the needs of stakeholders – can create the values of customization (Nath and Mukherjee, 2012). In the standpoint of service providers, structural bond refers to corporate efforts such as the introduction of policies and programs to design customized jobs. The right to make decisions regarding jobs such as business hours and business schedule by service providers is receiving customized values tailored to the needs of service providers from the corporation (Wang, 2014).

### Relationship Bonds and P-O Fit

Individuals’ attitude or behavior may change within organization based on whether there is a high similarity between individuals and organization or the degree of individual’s value and organization’s values coinciding with each other (Meglino and Ravlin, 1998). P-O fit, a part of person-environment fit, may be defined as the degree of compatibility between individuals and organization (Kristof, 1996). This is determined through how similar organization’s values and objectives are with individuals or how far organization can support individual’s values and objectives (Downes et al., 2017). When individual’s values and organization’s values are judged to be similar to each other, individual thinks that he/she is a part of organization (Saks and Ashforth, 1997), perceives organization’s goal as his/her goal and, pursues it with the strong sense of unity with organization (Cable and DeRue, 2002). On the contrary, when P-O fit is low, individual experiences psychological discomfort due to the discrepancy between the two different values and shows negative emotion and attitude (Lauver and Kristof-Brown, 2001). For this reason, previous studies identified P-O fit as the predisposing factor influencing job attitude of employees. P-O fit improves job satisfaction and organizational commitment by having positive influence on engagement of employees (Biswas and Bhatnagar, 2013) and lowers turnover intention (Liu et al., 2010). In particular, P-O fit not only lowers emotional exhaustion of service provider but also makes positive influence on occupational behavior of service provider (Yoo et al., 2014). Thus, it is important to understand some underlying factors which influence P-O fit of current employees.

Financial incentives not only motivate individuals but also become an element of values (Miao and Evans, 2007). Individuals interact with organization to obtain financial values. Individuals accomplish the goal organization wants and organization satisfies individuals’ financial needs. Through this continuous interaction process, individuals match their values and goals with organization (Abdalla et al., 2018). If individuals do not receive financial compensation which is proper to their performance in interaction with organization, individuals are likely to show negative response after feeling that it is unequal (Newman and Sheikh, 2012). On the other hand, individuals who have received plenty of financial values through interaction with organization feel guilty about the behaviors going against the interests of organization (Wang, 2014). This is the powerful evidence which shows that the values of service providers and the values of firm coincide with each other owing to financial values.

H1. Financial bonds will increase the P-O fit of service providers.

Powerful social interaction is helpful for work performance through relationship network between individuals or enables approaching essential information (Biong and Ulvnes, 2011). Furthermore, one can make joint interpretation with regard to certain phenomenon or situation by sharing and understanding one another’s vision, goal, and meaning to a high level (Tsai and Ghoshal, 1998). When this is expanded, the values of organization and the values of individuals coincide with each other through interaction between individuals. Service providers can make specific contribution through values and jobs of organization by sharing information through communication with colleagues. As examined so far, the socialization process within organization plays an important role in forming values of service providers so that they match corporate values (Kilroy et al., 2017).

H2. Social bonds will increase the P-O fit of service providers.

Structural bonds, providing customized organization system to resolve occupational problems of service providers, improves the relationship between corporation and service providers. Designing corporate system so that it enables flexible business schedule setting have service providers be immersed in corporation by providing customized values which are difficult to get from other corporation (Wang, 2014). Furthermore, structural bond plays the role of the sign of delivering corporate values and goals to service providers. High autonomy with regard to job is perceived as the message that corporation is convinced of individuals’ capabilities (Saragih, 2011). Thus, participation in corporate objective development increases and ownership and sharing of corporate values increase (Kilroy et al., 2017). Eventually, corporation can enhance P-O fit by providing the parts necessary in job to service providers and sharing similar characteristics through structural bonds (Kristof, 1996).

H3. Structural bonds will increase the P-O fit of service providers.

### P-O Fit and Emotional Labor

Previous studies identified that P-O fit not only had positive influence on individuals’ attitude and performance with regard to job (Downes et al., 2017; Abdalla et al., 2018) but also reduced negative factors such as burnout (Kilroy et al., 2017). Other emotional labor strategies are used in customer service context based on the degree of service providers perceiving P-O fit. When service providers perceive that their own values coincide with corporate values, they enthusiastically immerse themselves into job (Kim et al., 2013). In other words, since service providers take conducting emotional labor to accomplish corporate goals as one’s own role, service providers consciously try to revise their own emotions and internalize the emotions required by corporation. Thus, service providers earnestly express emotions through deep acting. On the contrary, service providers who experienced discord with organization undergo less positive or even negative emotions (Lauver and Kristof-Brown, 2001; Gabriel et al., 2014). Since service providers do not perceive their own values and corporate values as separate things, service providers are not motivated by the role corporations demands from them. Therefore, service providers can make positive expressions by suppressing or deceiving their emotions but do not revise emotions or make the effort to internalize display rules (Kilroy et al., 2017). Based on what has been examined so far, the following hypotheses have been set.

H4. P-O fit will reduce the surface acting of service providers.
H5. P-O fit will increase the deep acting of service providers.

### Moderating Effect of Collectivism

The studies on collectivism either examine the difference of value between countries with different cultures (Takano and Osaka, 1999) or are taking place on the aspect of exploring different tendencies of individuals (Arpaci et al., 2018). The purpose of this study is not comparing nations with different cultural values but examining how individual tendencies have varying influence on occupational aspects. Thus, this study aims to explore the effect of collectivism in terms of personal aspect. Collectivism has the characteristic of binding individuals through strict social framework and dividing them into in-group and out-group. Individuals display high loyalty toward in-group and expects that in-group will look after them (Hofstede, 1980). Service providers with strong collectivism put greater emphasis on relational aspect than their needs and make responses which are cooperative toward organization after perceiving relational behaviors as important (Ozdemir and Hewett, 2010). Thus, if service providers cannot match their objectives to organizational goals (Triandis and Gelfand, 1998) and cannot abide by the norms between organization and themselves, service providers come to have greater will to accomplish the goal desired by organization since they feel guilty (Li and Dant, 1997). Since individuals with collective values have interest toward harmonizing with organization (Mulki et al., 2015), they can match their own values with organization’s values faster and stronger. Service corporation aims to satisfy customers by delivering intangible services and emotional display rules of service providers are performed at this time. If service providers have a high collectivism value, they strive to more actively internalize emotional display rules required by corporation since individual values and corporate values are matched stronger. Thus, surface acting will be weakened and deep acting will be strengthened.

**FIGURE 1:**
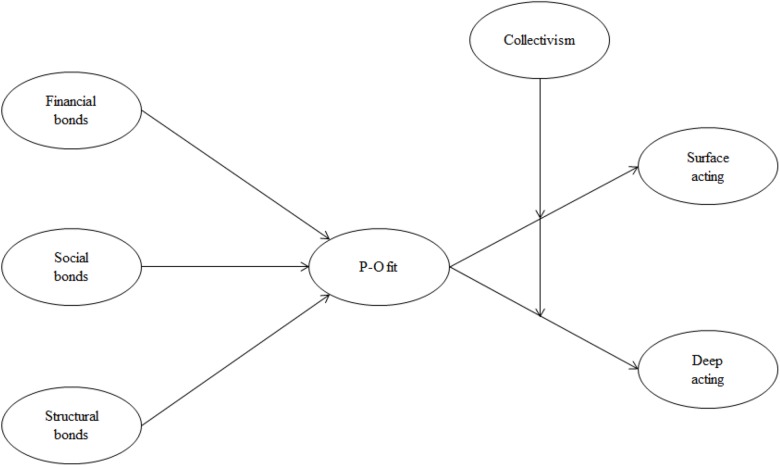
Research model.

H6. As the collectivism of service provider increases, the effect of P-O fit reducing surface acting will be strengthened.
H7. As the collectivism of service provider increases, the effect of P-O fit increasing deep acting will be strengthened. Based on the research hypotheses, we developed the final research model which is shown in the Figure 1.

## Methodology and Model Testing

### Participants and Procedure

This study collected data from 350 employees working in the financial industry in South Korea through online questionnaire survey panel and analyzed them. The employees are the leading service providers performing emotional labor while frequently coming into contact with customers. The corresponding panel has a lot of participants who agreed to take part in the study. The authors requested survey participants to choose their own occupation among several job categories and those who were not service providers were excluded in the survey. Then, respondents were asked about the industry they belong to and the survey was conducted for those who chose the financial industry.

The gender of the respondents was comprised of 195 men (55.7%) and 155 women (44.3%). When it came to age bracket, those in their 30s was the highest with 120 employees (34.3%) and it was followed by those in their 40s (109 employees, 31.1%), those in their 20s (80 employees, 22.9%), and those in their 50s (41 employees, 11.7%). When it came to work period, less than 3 years were 103 employees (29.5%), 3 years and longer and less than 6 years were 84 employees (24.0%), 6 years and longer and less than 9 years were 47 employees (13.4%), 9 years and longer and less than 12 years were 47 employees (13.4%), and 12 years and longer were 69 employees (19.7%). When it came to position, 178 respondents were staff (50.9%), 70 respondents were administrative managers (20.0%), 75 respondents were section chiefs (21.4%), and 27 respondents were general managers (7.7%).

### Measures

Relationship bond was assessed with ten items developed by Wang (2014). Financial bond was measured by three items. An example item is “My company provides satisfactory total income.” Cronbach’s alpha was 0.96. Social bond was measured by four items. An example item is “My company supports me so that I communicate well with co-workers.” Cronbach’s alpha was 0.88. Structural bond was measured by three items. An example item is “My company allows me to flexibly manage work schedules.” Cronbach’s alpha was 0.87. Correlations among the study constructs are displayed in the Table 1.

**Table 1:** Construct means, standard deviations, and correlations.

	1	2	3	4	5	6	7
1. Financial bonds	**0.94**						
2. Social bonds	0.24**	**0.87**					
3. Structural bonds	0.30**	0.30**	**0.81**				
4. P-O fit	0.50**	0.59**	0.50**	**0.85**			
5. Collectivism	0.43**	0.36**	0.26**	0.57**	**0.88**		
6. Surface acting	–0.02	0.11	–0.14*	0.04	0.16*	**0.81**	
7. Deep acting	0.18**	0.39**	0.38**	0.47**	0.25**	0.13*	**0.86**
Mean	2.57	3.60	3.38	3.11	3.07	3.36	3.64
*SD*	0.99	0.67	1.01	0.71	0.75	0.74	0.63


*The bolded value in the diagonal is the square root of the AVE; ^∗^p < 0.05, ^∗∗^p < 0.01.*

P-O fit was assessed with four items developed by Saks and Ashforth (2002). An example item is “To what extent are the values of the organization similar to your own values?” Cronbach’s alpha was 0.88. Collectivism was assessed with three items developed by Wu (2006). An example item is “Group success is more important than individual success.” Cronbach’s alpha was 0.87.

Emotional labor was assessed with eight items developed by Diefendorff et al. (2005). Surface acting was measured by four items. An example item is “I fake the emotions I show when dealing with customers.” Cronbach’s alpha was 0.85. Deep acting was measured by four items. An example item is “I try to actually experience the emotions that I must show to customers.” Cronbach’s alpha was 0.86.

All items were scored on a five point scale, ranging from 1 = strongly disagree to 5 = strongly agree. Measurement scales of the study constructs are shown in the Appendix.

### Convergent and Discriminant Validity of Constructs

Prior to verifying hypotheses, this study identified convergent validity and discriminant validity of constructs through confirmatory factor analysis (Nunnally, 1978; Fornell and Larcker, 1981). Convergent validity was identified by composite reliability (CR) and average variance extracted (AVE). The evaluation standard of CR is 0.7 and higher and the evaluation standard of AVE is 0.5 and higher. In a bid to identify discriminant validity, whether the square root value of AVE exceeded the correlations between constructs was examined. Since the fit of confirmatory factor analysis was shown as χ^2^ = 574.59(df = 254, *p* = 0.00), GFI = 0.88, CFI = 0.95, NFI = 0.91, RMSEA = 0.06, they were generally acceptable. Also, since the standards which could evaluate convergent validity exceeded the standard level, convergent validity was identified. As a result of identifying discriminant validity, the square root value of AVE with regard to constructs had been found to exceed the correlations between constructs. Therefore, discriminant validity had been found to have no problems.

### Common Method Bias

Since self-reported measures are utilized, there is a danger of common method bias. In their argument of the causes and complications of this bias, Podsakoff et al. (2003) suggested both procedural antidotes, such as acquiring measures from different respondents or methodologically disconnecting measures, and statistical antidotes to diagnose the bias. This study adopts Harman’s single factor test that Podsakoff et al. (2003) announced as a statistical remedy. Adopting exploratory factor analysis, the number of factors and variances inferred from the unrotated factor solutions are tested. The analysis identifies seven factors with an eigenvalue greater than 1; their variances are 30.92, 12.30, 10.27, 7.83, 6.02, 5.69, and 4.05%. As the analysis identifies more than one factor and the factor with the highest variance elucidate less than 50% of the total variance, it can be considered that the study does not have a serious common method bias.

### Test of Hypotheses

**FIGURE 2:**
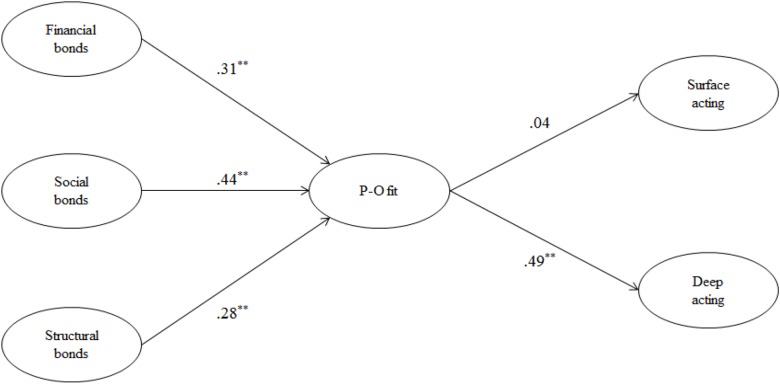
Results of hypotheses testing. (χ^2^ = 529.94, df = 201, *p* = 0.00, GFI = 0.88, CFI = 0.94, TLI = 0.93, NFI = 0.90, RMSEA = 0.07). ^∗∗^*p* < 0.01.

Since the fit of this study model was χ^2^ = 529.94(df = 201, *p* = 0.00), GFI = 0.88, CFI = 0.94, TLI = 0.93, NFI = 0.90, RMSEA = 0.07, it is generally at an acceptable level. Figure 2 is the result of verifying hypotheses through structural equation model. This study expected that relationship bonds will have positive influence on P-O fit of service providers. As a result of identifying the hypotheses, relationship bonds elements – financial bonds (β = 0.31, *p* < 0.01), social bonds (β = 0.44, *p* < 0.01), and structural bonds (β = 0.28, *p* < 0.01) – had positive influence on P-O fit. Thus, H1, H2, and H3 were supported. Next, this study expected that P-O fit will have negative influence on surface acting and positive influence on deep acting. As a result of identifying the hypotheses, P-O fit did not have any influence on surface acting (β = 0.04, *p* > 0.05) but had positive influence on deep acting (β = 0.49, *p* < 0.01). Therefore, Hypothesis 4 was rejected but Hypothesis 5 was supported.

### Test of Moderating Effect

**FIGURE 3:**
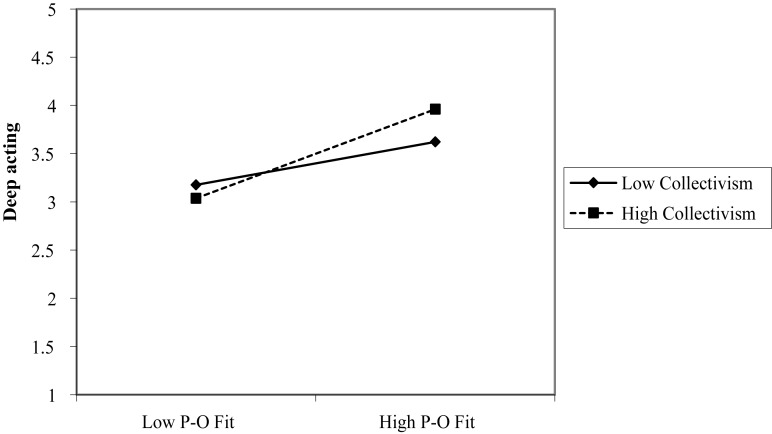
Interactive effects of collectivism and P–O fit on deep acting.

This study aimed to identify the moderating effects of collectivism between P-O fit of service providers and emotional labor. Since P-O fit had been found to not have any influence on surface acting, the moderating effect toward deep acting was identified. In order to identify moderating effects, interaction effect was verified through the process model using bootstrap (Preacher and Hayes, 2008; Hayes, 2013). Hayes (2013) used No.1 model out of the suggested process models, Y inserted deep acting, X inserted P-O fit, and M inserted collectivism. In addition, education, work period, position and income of service providers were controlled. As a result of the analysis (Table 2), the interaction effect of collectivism was statistically significant with regard to deep acting (β = 0.1197, CI = [0.0040, 0.2354], *p* < 0.05). In a bid to specifically identify moderating effect, *post hoc* test was conducted. For this aim, low level simple slope (-1 SD) and high level simple slope (+1 SD) were identified from the average of collectivism (Aiken and West, 1991). As a result of the *post hoc* test, as shown in Figure 3, the influence by P-O fit on deep acting increased as collectivism of service providers went higher. Thus, Hypothesis 7 was supported.

**Table 2:** Results of moderating effects of collectivism.

Variable	Bias-corrected bootstrap 95% confidence interval				
	
	Coeff.	s.e.	*p*	CI_low_	CI_high_
**DV = DEEP ACTING**		
Main effects: P-O fit	0.3429	0.0583	0.0000	0.2282	0.4575
Collectivism	0.0499	0.0568	0.3801	–0.0618	0.1616
Interactions: P-J fit × Collectivism (H 7)	0.1197	0.0588	0.0427	0.0040	0.2354
Controls: Education	–0.0223	0.0325	0.4932	–0.0862	0.0416
Work period	0.0458	0.0278	0.1003	–0.0089	0.1006
Position	–0.0285	0.0432	0.5096	–0.1135	0.0565
Income	0.0624	0.0328	0.0583	–0.0022	0.1269
(High group) P-J fit × Collectivism	0.4323	0.0713	0.0000	0.2921	0.5725
(Mean group) P-J fit × Collectivism	0.3429	0.0583	0.0000	0.2282	0.4575
(Low group) P-J fit × Collectivism	0.2535	0.0747	0.0008	0.1066	0.4003


*Process model 1; N = 350; Bootstrap samples 5,000.*

## Conclusion and Discussion

This study examined relationship bonds for corporation to form relationship with service providers. This study developed and tested the model which identified the influence on emotional labor by relationship bonds through P-O fit and the role of collectivism. This study discovered the following findings. Three elements of relationship bonds – financial bonds, social bonds, and structural bonds – are positively related to deep acting through the increase of P-O fit. The influence on deep acting by P-O fit is moderated by collectivism of service provider. Specifically, as collectivism of service provider goes higher the effect of P-O fit increasing deep acting becomes stronger.

### Theoretical Implications

There are several theoretical contributions. First, it contributed to relationship marketing literature by applying the concept of relationship bonds into service providers. Owing to the influence by service providers regarding customers, the importance of forming relationship between corporation and service providers is being emphasized (Tortosa et al., 2009). Relationship marketing literature found that relationship bonds is the strategy for forming relationships and the antecedent factor for accomplishing relationship performance (Dash et al., 2009). Previous studies identified relationship bonds as the strategy to maintain relationship between corporation and customers or corporations (Wang, 2014) but the studies on service providers merely stay at initial level. Moreover, the studies have identified that performances changing owing to the ways corporation handle service providers but academic discussion on forming relationship between corporation and service providers is not taking place (Herington et al., 2006). This study aimed to overcome the limitation of previous studies by examining relationship bonds from the aspect of service providers.

Second, this study suggested the empirical evidence identifying the process of relationship bonds influencing emotional labor. Since service providers directly perform emotional labor to customers, it is important to identify the factors influencing emotional labor of service providers. The previous studies on emotional labor treated individual characteristics – personality traits, emotional abilities, and so on – and event characteristics – moods, customer mistreatment, and so on – as major predisposing factors (Grandey and Gabriel, 2015). However, there was no empirical evidence identifying how relationship bonds, forming relationship between corporation and service provider, was related to emotional labor. This study suggested relationship bonds as the major previous study having influence on emotional labor.

Third, owing to relationship bonds, this study identified the roles of P-O fit when emotional labor response is occurring. This study provides the understanding of the mechanism explaining how environmental factors of service providers affect emotional labor. Emotional labor is an essential element for service providers and this implies that it is crucial for corporations, which have to induce positive emotional labor strategies from service providers, to give support so that individuals feel conformity with organization. Almost all service sites require emotional labor (Tschan et al., 2005). Therefore, understanding of P-O fit roles in service jobs is a good way of carrying out positive emotional labor strategies in service industry which frequently interacts with customers.

Fourth, this study was expanded since the moderating effect of collectivism between P-O fit and emotional labor was identified. Previous studies suggested the moderating role of collectivism from cultural psychology aspect with regard to work behavior and performance of employees (Hui et al., 2015; O’Neill et al., 2015). However, specific role between fit and emotional labor was not mentioned. This study suggested clear and specific viewpoint explaining emotional labor from cultural psychology aspect by identifying the moderating effect of collectivism between P-O fit and emotional labor.

### Practical Implications

Through the results of this study, several practical implications can be discussed. First, corporations can improve P-O fit of service providers through relationship bonds. Since P-O fit enhances performance by being positively applied to service providers (Downes et al., 2017; Abdalla et al., 2018), it is important to manage relationship bonds. Corporate financial support becomes the element of value to individuals and acts positively toward accomplishing organization’s goals since individuals equate themselves and organization (Miao and Evans, 2007). Therefore, corporations need to form financial bonds by establishing financial compensation system which service providers perceive as sufficient. In addition, corporations need to come up with the measures to improve the social relations among service providers. Communication among service providers improves the ability or skills to resolve problems by promoting information sharing (Felício et al., 2014). Thus, corporate efforts to establish positive relationship among service providers by invigorating corporate communities and organizing horizontal corporate culture are necessary. Through these efforts, social sense of belonging by service providers can also be fulfilled. Structural bond is a positive factor toward P-O fit. The system of service providers flexibly adjusting one’s own work schedule assists the balance between work and life and immerses workers into jobs (Martin and MacDonnell, 2012). Since corporate efforts to form structural bonds plays the role of resources in service providers doing work, the efforts not only appear as performance by being connected to work efficiency improvement but also reduces the expenses arising from stress (Zablah et al., 2012).

Second, P-O fit can improve deep acting of service providers. Corporation needs to interact with service providers to deliver the goals and values pursued by corporations. Corporations can induce positive behaviors by enhancing similarity between corporation and service providers (Meglino and Ravlin, 1998). Since deep acting by service providers lets customers to experience high service quality after delivering sincere emotions (Pugh, 2001; Hennig-Thurau et al., 2006), relationship bonds strengthens deep acting through P-O fit and is positively related to performance. Meanwhile, the influence by P-O fit on surface acting was not significant. Surface acting, service providers expressing their emotions to customers after controlling and adjusting them, appears in the interaction with customers. Therefore, the negative factors related to customers such as customer incivility have direct influence on surface acting (Sliter et al., 2010). Since P-O fit is not the factor related to direct interaction with customers, it cannot influence surface acting.

Third, collectivism increases the influence by P-O fit with regard to deep acting of service providers. This study suggested that corporation managers must take into account cultural values. In particular, since corporate global management increases, managers must consider countries’ cultural backgrounds and values. This can provide better insight and understanding regarding employment, arrangement, and education of service providers when it comes to service firms entering new markets. While Asian countries like Korea can use the method of hiring collective individuals as the corporate measure to manage emotional labor, U.S. has limited number of applicants with collective values (Astakhova, 2016). Therefore, service firms must apply management styles suiting cultural norms so as to accomplish maximum results by hiring local talents.

### Limitations and Future Direction of Study

Even though this study suggested theoretical and practical implications, it has several limitations. First, this study examined the factors of relationship bonds into three aspects: financial bonds, social bonds, and structural bonds. Service providers border between corporation and customer owing to occupational characteristics. Thus, social bonds has to be examined by dividing into social bonds of internal aspect related to corporation and social bonds of external aspect related to customer. However, this study examined social bonds only in the aspect related to corporation. The scope of study will be expanded further if social bonds is divided into two aspects.

Second, this study identified that relationship bonds act positively in deep acting through P-O fit of service providers. It can be expected that performance will improve if deep acting increases but this study did not reflect performance aspect. Future studies need to suggest more completed study model by taking into consideration performance aspect of service corporations.

Third, this study collected data targeting service providers in the financial industry. The employees in the financial industry have the characteristics distinct from other industries. For instance, services dealt by employees in the financial industry are more complicated than other service industries. This may make applying this study results into the entire service industries difficult. Therefore, the generalization of this study results has to be induced by conducting studies targeting diverse industries.

Fourth, this study identified which organizational level factors influence emotional labor through P-O fit of current employees. It is also important to identify the effects of job characteristics on emotional labor through P-O fit and therefore, future study needs to investigate how P-O fit can be influenced by job characteristics of current employees.

## Ethics Statement

This study was carried out in accordance with the recommendations of ethics committee of Hanyang University with written informed consent from all subjects. All subjects gave written informed consent in accordance with the Declaration of Helsinki. The protocol was approved by the ethics committee of Hanyang University.

## Author Contributions

M-SL designed the work and analyzed the data. S-LH designed and drafted the work. SH collected and analyzed the data. HH collected and analyzed the data.

## Conflict of Interest Statement

The authors declare that the research was conducted in the absence of any commercial or financial relationships that could be construed as a potential conflict of interest.

## Appendix

**Table A1:** Items and CFA results.

Construct	Items	λ^a^	α	CR^b^	AVE^c^
Financial bonds	1. My company provides satisfactory total income.	0.94	0.96	0.95	0.88
	2. My company provides satisfactory monthly salary.	0.98			
	3. My company provides anticipated wage.	0.90			
Social bonds	1. My company supports me so that I communicate well with co-workers.	0.81	0.88	0.93	0.76
	2. My company supports me so that I maintain good relationships with co-workers.	0.80			
	3. My company supports me so that I communicate well with my boss.	0.83			
	4. My company supports me so that I maintain a good relationship with my boss.	0.80			
Structural bonds	1. My company allows me to decide the amount of extra work.	0.68	0.87	0.85	0.66
	2. My company allows me to freely adjust working hours.	0.91			
	3. My company allows me to flexibly manage work schedules.	0.90			
P-O fit	1. To what extent are the values of the organization similar to your own values?	0.79	0.88	0.91	0.72
	2. To what extent does your personality match the personality or image of the organization?	0.82			
	3. To what extent does the organization fulfill your needs?	0.79			
	4. To what extent is the organization a good match for you?	0.80			
Collectivism	1. Group welfare is more important than individual rewards.	0.79	0.87	0.91	0.77
	2. Group success is more important than individual success.	0.93			
	3. Employees should pursue their goals after considering the welfare of the group.	0.78			
Surface acting	1. I put on an act in order to deal with customers in an appropriate way.	0.63	0.85	0.88	0.66
	2. I fake a good mood when interacting with customers.	0.77			
	3. I put on a “mask” in order to display the emotions I need for the job.	0.90			
	4. I fake the emotions I show when dealing with customers.	0.76			
Deep acting	1. I try to actually experience the emotions that I must show to customers.	0.81	0.86	0.92	0.74
	2. I make an effort to actually feel the emotions that I need to display toward others.	0.84			
	3. I work hard to feel the emotions that I need to show to customers.	0.81			
	4. I work at developing the feelings inside of me that I need to show to customers.	0.69			
χ^2^ = 574.59, df = 254, *p* = 0.00, GFI = 0.88, CFI = 0.95, TLI = 0.93, NFI = 0.91, RMSEA = 0.06		

*^*a*^All factor loadings are significant (p < 0.01).*

*^*b*^CR, composite reliability.*

*^*c*^AVE, average variance extracted.*
